# A Numerical Method to Model Non-linear Damping Behaviour of Martensitic Shape Memory Alloys

**DOI:** 10.3390/ma11112178

**Published:** 2018-11-03

**Authors:** Pouya Haghdoust, Antonietta Lo Conte, Simone Cinquemani, Nora Lecis

**Affiliations:** Politecnico di Milano, Department of Mechanical Engineering, Via La Masa 1, I-20154 Milan, Italy; antonietta.loconte@polimi.it (A.L.C.); simone.cinquemani@polimi.it (S.C.); nora.lecis@polimi.it (N.L.)

**Keywords:** SMA, damping, nonlinear

## Abstract

This article investigates the efficiency of hybridizing composites with thin layers of martensitic shape memory alloys for improvement of damping. The non-linear damping behaviour of martensitic shape memory alloys is simulated using a modified version of Masing’s rules. The model was implemented in a user subroutine of a finite element code, and validated by a numerical simulation of experimental hysteresis loops at different maximum strain amplitudes. The experimental free decay of hybridized glass fiber reinforced polymer beams was simulated using the finite element model, including the validated model of the investigated materials. The amplitude-dependent damping of the hybrid beams in free decay was reproduced successfully in the numerical analysis and it was proven that the hybridization technique is efficient for improvement of damping.

## 1. Introduction

Shape Memory Alloys (SMA) are being used extensively for different purposes in this decade and the research projects on them have a broad range, varying form metallurgical issues involved in their damping mechanisms to research which investigates the technologies for SMA joining [1,2]. Also, their application field ranges from hybridizing techniques in composite structures in order to customize specific material performances, to more advanced fields such as micro actuation systems. In many studies, they have been used for vibration control purposes by employing an active or a passive strategy [3,4,5,6].

A comprehensive review on this issue is available in [7]. In [8] Birman illustrated a two-step approach in the strength and stiffness analyses of fibre reinforced particulate-matrix composites obtained through a generalization of available micromechanical solutions available for three-phase materials. The numerical analysis shows that adding stiff particles to the matrix results in a significant enhancement of the transverse strength and stiffness. Balapgol et al. [9] investigated the natural frequencies of a multilayer SMA laminated composite cantilever plate identifying the thickness of the SMA layer, the position of the SMA layer, the temperature of the SMA and the span-to-depth ratio all playing important roles in controlling the free vibration of the SMA/elastomer actuator. The advantages of using the damping capacity of thermoelastic martensite in shape memory alloys is discussed in [10]. In [11,12,13,14] martensitic SMA sheets were embedded into Glass Fiber Reinforced Polymer (GFRP) beams to passively enhance the damping of the system. While the concept of hybridization for enhancing the damping of the structures dates back to 40 years ago [15], SMA hybridized systems are considered as newcomers in this field. However, since these materials receive more attention in the field of vibration control, more suitable numerical models are required in order to study the damping behaviour of material and systems integrated with SMA materials.

**Figure 1:**
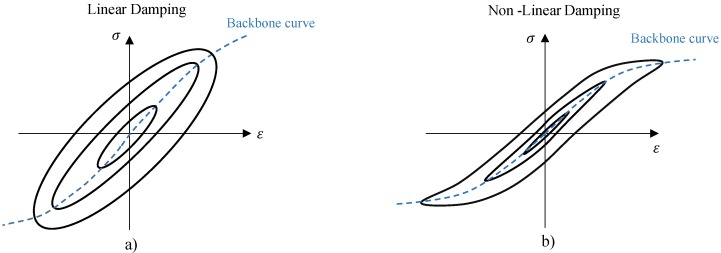
Schematic of a hysteresis cycle for materials with: (**a**) linear and (**b**) nonlinear damping.

**Figure 2:**
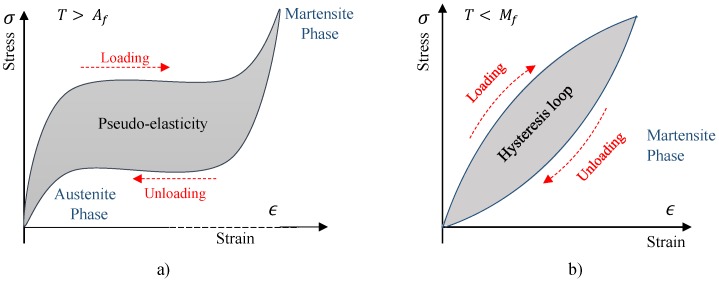
(**a**) Schematic of a pseudo-elastic behaviour. (**b**) Schematic of stress strain nonlinearity at low strain ranges (less than 0.4%) in the martensitic state of a SMA alloy.

Regardless of the different physical mechanisms involved, all real materials dissipate some level of energy, no matter how little, during cycling deformation. Generally, such effect is highly nonlinear and related to many factors such as temperature, frequency, strain, and strain rate, while the linear behaviour assumption has only a limited application. A plot of instantaneous stress vs instantaneous strain, for all values of time during a steady-state of forced vibration tests, is referred to as a hysteresis cycle and is a well established experimental approach in classifying and quantifying the internal damping behaviour of materials. Metals alloys, as well as severe high damping alloys, show elliptical hysteresis cycles with linear damping, including viscous damping, hysteretic damping, and linear rate depending damping, while the hysteresis cycles show a more sophisticated shape with a nonlinear damping (Figure 1) [16]. In case of SMA materials, frequency and amplitude dependencies of damping dependencies have been reported in many studies [12,17]. The intrinsic amplitude-dependent damping is associated to two different phenomena: pseudo-elasticity and the dissipation of energy in the martensitic state (Figure 2). The former occurs in SMA materials with an austenitic phase at their operating regime, where the stress-induced martensite will generate the pseudo-elastic behaviour of the material, while the latter occurs in the SMAs with a martensitic microstructure at operating regime. The high damping values reported in this case were ascribed to the high density of mobile twins and mobile interfaces [10].

There are several numerical and experimental studies in the literature which investigate the damping on the basis of a pseudoelastic behaviour of SMAs. Micromechanical models which consider phase transformation are extensively used in this case [18,19,20,21,22]. Nonetheless, stable martensitic microstuctural conditions have more practical advantages for applications with limited stress and strain fields, such as the application of the hybridized composites for passive damping [10], where the phase transformation could cause micro-damage in the composite matrix.

The damping in the martensitic state has received less attention and accurate models are not available. With the aim to model amplitude-dependent damping of SMA in martensitic phase, and to use this model in numerical analysis of component made by hybrid composite [23], a classical approach to model material damping is proposed. The techniques available in the literature in order to model the material damping are usually limited to a linearity assumption. Adams and Bacon [24] illustrated a damping evaluation process in which energy dissipation was described as separable dissipations inside components. This was then developed and used in other works such us [25,26]. In the case of high damping materials or large structures with high amplitude vibrations the amplitude-dependent damping cannot be neglected and the linear assumption is no longer accurate. In [27] the nonlinear forced vibration analysis of laminated composite beams is investigated by developing numerical methods. In [28] four different damping models are compared, and a solid model of linear viscoelasticity is proposed, as it is more suitable for investigating the nonlinear damping.

This study applied a methodology based on modelling nonlinearity damping through the modelling of a material’s hysteresis behaviours. A phenomenological model, based on a modified version of Masing’s rules, has been developed to reproduce SMA’s hysteresis cycle at low strain ranges when they are in a martensitic state. The model was implemented in a user material subroutine for the Abaqus finite elements commercial code. To validate the material model, the hysteresis cycles reproduced numerically were compared with the hysteresis cycles obtained through experiments at different maximum strain levels. Finally, the validated model was used to simulate the free decay of SMA/GFRP hybrid beams.

## 2. Materials

The SMA alloys under study are Ni_40_Ti_50_Cu_10_ and Cu_66_Zn_24_Al_10_. They were produced previously in the laboratory by means of a vacuum induction using high purity metal powders [11,12]. The transformation temperatures, through the Differential Scanning Calorimetry (Q100 DSC, TA Instruments, New Castle, England) are reported in Table 1. Both alloys are in a martensitic phase at ambient temperature.

The damping of these materials was investigated through cyclic tensile tests which were performed on sheets sized 200 mm × 20 mm × 0.2 mm at room temperature and at a different maximum strain amplitude, with an MTS hydraulic machine equipped with a 5 kN load cell. The strain measurements were performed using a 50-mm extensometer gauge length at 0.05 Hz. More details on the experimental tests are reported in [11,12].

**Figure 3:**
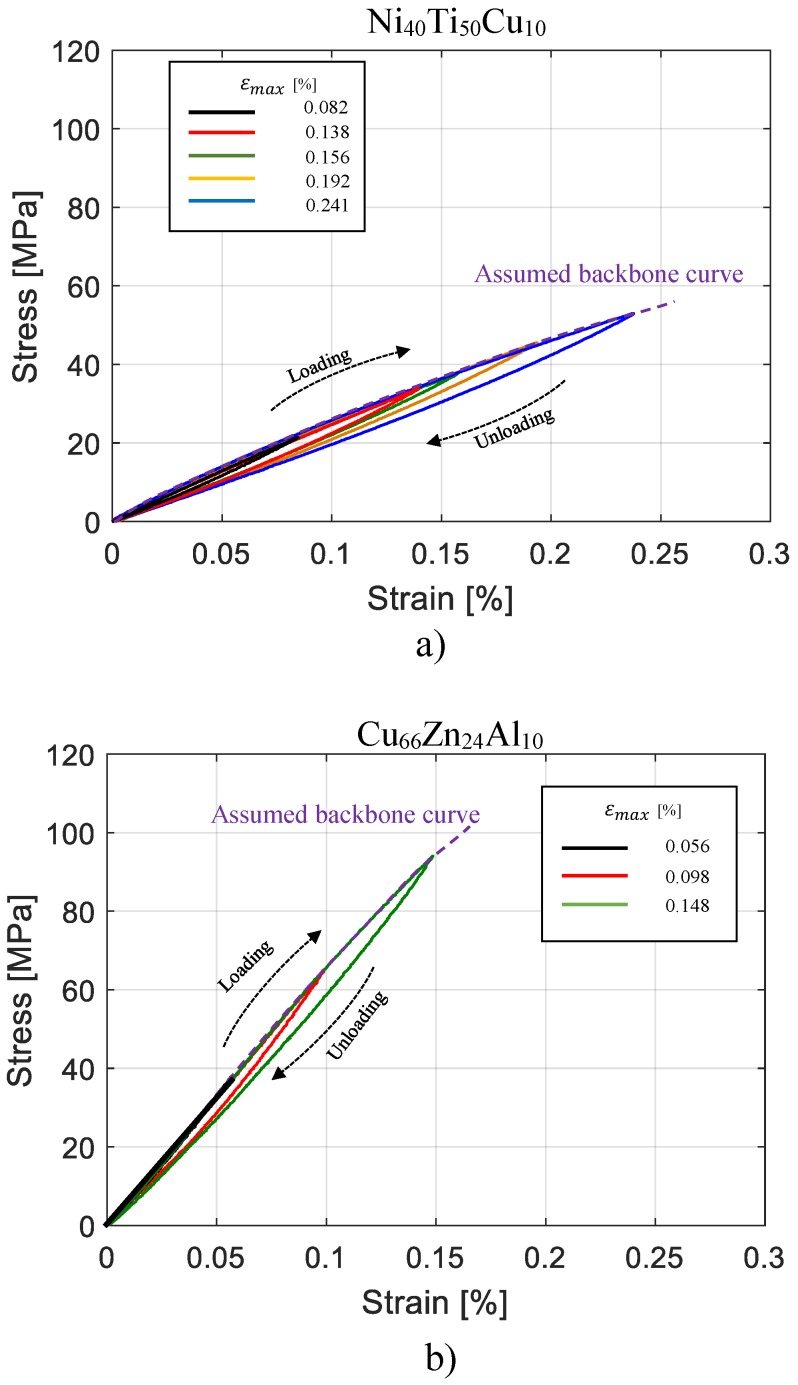
Nonlinear stress–strain behaviour of the materials investigated at different strain amplitudes: (**a**) NiTiCu, (**b**) CuZnAl.

Figure 3 shows the nonlinear stress–strain behaviour of the investigated materials at different strain amplitudes. As assumption to formulate the damping model based on hysteresis cycles, at each series of test at new value of maximum strain amplitude the irrecoverable strain observed before the cycle stabilization has been neglected. The investigated amplitude range for two different materials are determined from the strain amplitude range observed in vibrating condition as they are embedded in a beam shape composite structure as reported in [11,12]. As the elastic modulus of Ni_40_Ti_50_Cu_10_ is lower than the elastic modulus of Cu_66_Zn_24_Al_10_, the strain range investigated in the case of Ni_40_Ti_50_Cu_10_ is higher than the one of Cu_66_Zn_24_Al_10_. Both Ni_40_Ti_50_Cu_10_ and Cu_66_Zn_24_Al_10_ alloys exhibit a high dependency of the total dissipated energy on strain, whereas the dependency on the frequency is much smaller and can be neglected. The nonlinear behaviour observed accounts for a high level of dissipated energy, in contrast with the small strain nonlinearity, also observed in typical constructional metals [29], but associated with extremely thin loading–unloading loops. Moreover, the experimental hysteresis cycles provide the following relevant evidence:

- at each maximum strain level the cycle is fully reversible and reproducible at each maximum strain level;
- the cyclic loading path always follows the backbone curve;
- each new series of cycles at a given maximum strain is not affected by a previous series of cycles at different maximum strain levels.

Recently, this type of hysteresis has been classified as kinking nonlinear elastic hysteresis [11,30,31]. For a large class of solids, including graphite, titanium, magnesium, cobalt, and sapphire, it has been attributed to the formation of dislocation-based incipient kink bands with multiple parallel dislocation loops, where dislocations segments of opposite signs are present on either side. The idea related to the incipient kink bands is that there is a threshold stress needed to nucleate them and removing the load results in their spontaneous collapse and a return of the microstructure to the virgin state.

With regard to the materials investigated, there is no experimental evidence that this mechanism may be the physical origin of the energy dissipation, except that the actual coarse grained microstructure dissipates more than the previously investigated fine-grained counterparts, and a non-cyclic softening is observed after more than 100 cycles at the same maximum strain amplitude.

This study used the experimental hysteresis cycles with related properties, for Ni_40_Ti_50_Cu_10_ and Cu_66_Zn_24_Al_10_ alloys, with the aim to model the nonlinear and frequency independent damping for a small strain range (less than 0.4%) and moderate range of frequencies regardless of the internal mechanisms [32].

## 3. Model of the Nonlinear Damping Behaviour

To model the nonlinear damping, viscoelastic material models such as Kelvin–Voigt, a standard linear solid or Boltzmann’s models were used [28]. Although the mentioned models can model a high dependency of the total dissipated energy on the strain, their sometimes poor accuracy and difficulty to identify the model parameters are drawbacks. Few researchers have focused on the use of phenomenological nonlinear damping models. Gottlieb and Habib [33] used a phenomenological nonlinear damping model to understand the large amplitude vibrations of a spherical pendulum. Eichler et al. [27] used a damping model containing a nonlinear term proportional to the square of the vibration amplitude multiplied by the velocity. Recently, Amabili [34] derived a nonlinear damping model based on a fractional standard linear solid material.

**Figure 4:**
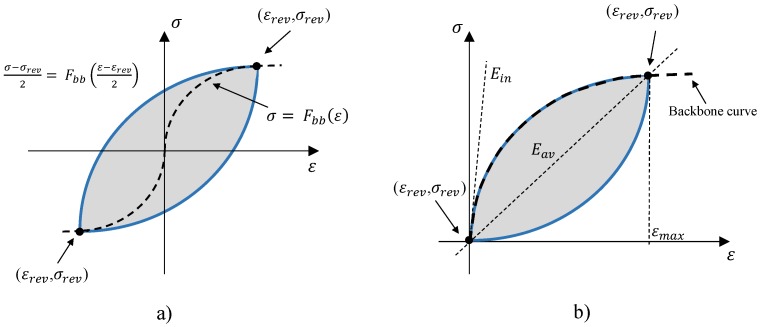
(**a**) Masing’s rule for an irreversible stress–strain behaviour. (**b**) Schematic of the reference hysteresis cycle of reference for the proposed hysteresis function.

In this study, with the aim to model an amplitude-dependent damping for the materials investigated, a frequency-independent phenomenological model for elastic hysteresis was developed and fitted with the experimental results. The developed model is a modified version of Masing’s theory as the principal hysteresis rule. Masing’s rules were originally introduced in 1926 [35] and then extended by Karmer [36] in 1966 to four statements (Figure 4a):

1. For an initial loading in a cyclic test, the stress strain path follows the backbone curves:
  (1) $$\sigma =F_{bb}\left(\varepsilon \right)$$
  where $F_{bb}\left(\varepsilon \right)$ is called backbone function.
2. If a stress reversal occurs at a point defined by ($\varepsilon_{rev}$, $\sigma_{rev}$), the stress strain path will be given by:
  (2) $$\frac{\sigma -\sigma_{rev}}{2}=F_{bb}\left(\frac{\varepsilon -\varepsilon_{rev}}{2}\right)$$
3. If the loading curve intersects the backbone curve, it follows the backbone curve until the following stress reversal.
4. If an unloading or reloading curve crosses an unloading or reloading curve from the previous cycle, the stress–strain curve follows that of the previous cycle.

A modified formulation of Masing’s second rule was suggested by G. Muravskii [37] as:

(3) $$\sigma -\sigma_{rev}=\varphi \left(\varepsilon -\varepsilon_{rev}\right)$$

where φ is called hysteresis function.

Different researchers have introduced in literature several hysteresis functions, each of which proposed particular behaviours [32,38]. For example, the one introduced by Puzrin and Burland [39] and the one proposed by Pyke [40], where cycles become symmetrical after an increase in their number, are suitable for describing the behaviour of granular materials. All the mentioned cases are developed for materials with non-elastic hysteresis behaviours, relating to irreversible physical mechanisms. The parameters of the hysteresis functions are obtained on the basis of $F_{bb}$ function, calculated by using the initial loading curve. Consequently, none of the mentioned material models can be implemented to reproduce the nonlinear damping behaviour associated with an elastic hysteresis cycle as observed in Ni_40_Ti_50_Cu_10_ and Cu_66_Zn_24_Al_10_ alloys.

As mentioned, in the case of elastic-hysteresis cycles, the initial loading curve (backbone curve) is identical to the loading path of the hysteresis cycle, and a distinguished backbone curve is not available. To solve this problem, the authors have proposed the following hysteresis function:

(4) $$\varphi =\frac{\left(E_{av}-E_{in}\right)}{\varepsilon_{max}}{\left(\varepsilon -\varepsilon_{rev}\right)}^{2}+E_{in}\left(\varepsilon -\varepsilon_{rev}\right)$$

where the parameters $E_{av},E_{in},\varepsilon_{max}$ represent, respectively, the average elastic modulus, the initial elastic modulus, and the maximum strain (See Figure 4b) of the hysteresis cycle available with the highest amplitude, which hereafter is mentioned as hysteresis of reference. The proposed function guarantees lens shaped cycle as observed in the cyclic tensile tests for the materials investigated (Figure 3). For a fixed $\varepsilon_{max}$ higher value of $E_{av}-E_{in}$ would result in larger hysteresis cycles and a consequent higher damping value. Considering Equation (4) as the hysteresis function, the elastic modulus of the material for each strain increment would be equal to:

(5) $$E_{\left(i\right)}=\frac{\Delta \sigma_{\left(i\right)}}{\Delta \varepsilon_{\left(i\right)}}=\frac{2(E_{av}-E_{in})}{\varepsilon_{max}}\left(\varepsilon_{p,(i)}-\varepsilon_{rev}\right)+E_{in}$$

To model the behaviour observed for the investigated materials, using the proposed model, a user subroutine SMA-UMAT.for of Abaqus Finite Element (FE) commercial code was developed, and following rules were implemented:

- Hook’s law which related stress to strains for elastic isotropic materials in a plane stress condition was implemented to calculate stress at each increment (Equations (6)–(9)).
  (6) $$\left[\begin{array}{c} \sigma_{1}\\ \sigma_{2}\\ \tau_{12} \end{array}\right]=\left[\begin{array}{ccc} Q_{11} & Q_{12} & 0\\ Q_{12} & Q_{11} & 0\\ 0 & 0 & Q_{33} \end{array}\right]\left[\begin{array}{c} \varepsilon_{1}\\ \varepsilon_{2}\\ \gamma_{12} \end{array}\right]$$
  (7) $$Q_{11}=\frac{E_{\left(i\right)}}{1-\nu_{12}^{2}}$$
  (8) $$Q_{12}=\frac{\nu_{12}E_{\left(i\right)}}{1-\nu_{12}^{2}}$$
  (9) $$Q_{33}=G_{21}=\frac{E_{\left(i\right)}}{2(1+\nu_{12})}$$
- The actual elastic properties for each strain increment, were calculated according to Equation (5), based on the principal strain with highest absolute value ($\varepsilon_{p,(i)}$) and the last reversal strain ($\varepsilon_{rev}$). The actual principal strain is calculated from the strain values in standard direction ($\varepsilon_{1},\varepsilon_{1},\gamma_{12}$) at the beginning of the increment.
- Reversal points were detected from the sign change of the actual and previous principal strain and principal strain increments. In both cases the principal strain with highest absolute value is considered. Taking this into consideration results in the symmetrical behaviour of the model with respect to the origin of the axes.

**Figure 5:**
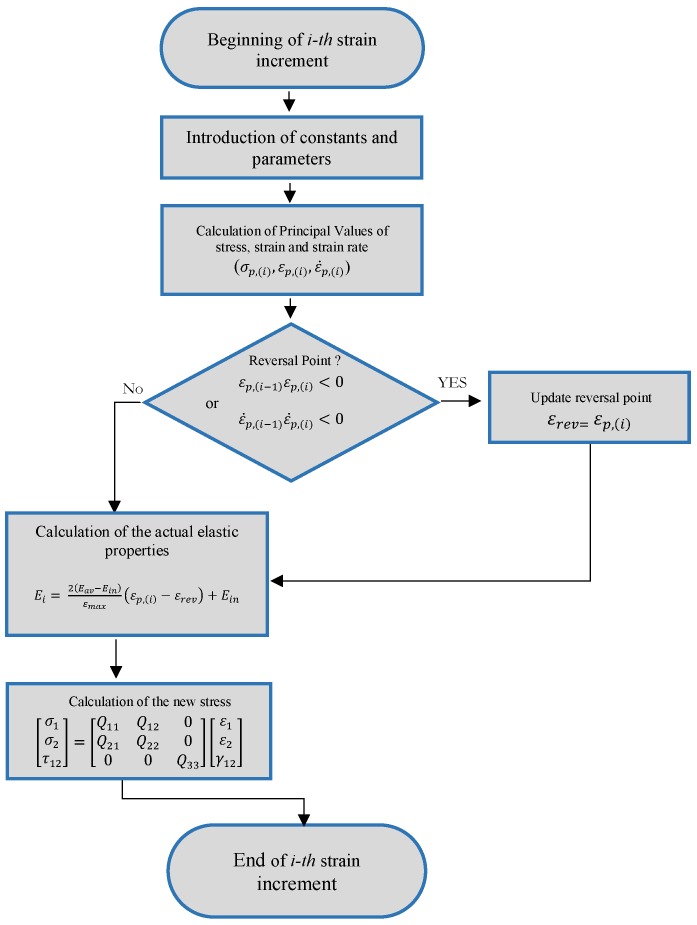
Flowchart of the SMA-UMAT.for subroutine.

The flowchart of the subroutine developed is presented in Figure 5.

## 4. Validation of the Material Model

**Figure 6:**
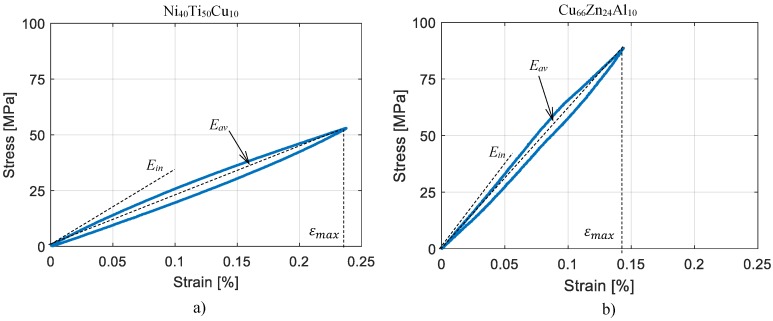
Hysteresis cycles of reference: (**a**) Ni_40_Ti_50_Cu_10_, (**b**) Cu_66_Zn_24_Al_10_.

The parameters of the modified Masing model (Equation (4)) were identified through the experimental reference hysteresis cycles presented in Figure 6 which corresponds to the largest cycles in Figure 3, for Ni_40_Ti_50_Cu_10_ and Cu_66_Zn_24_Al_10_, respectively, and are reported in Table 2.

**Figure 7:**
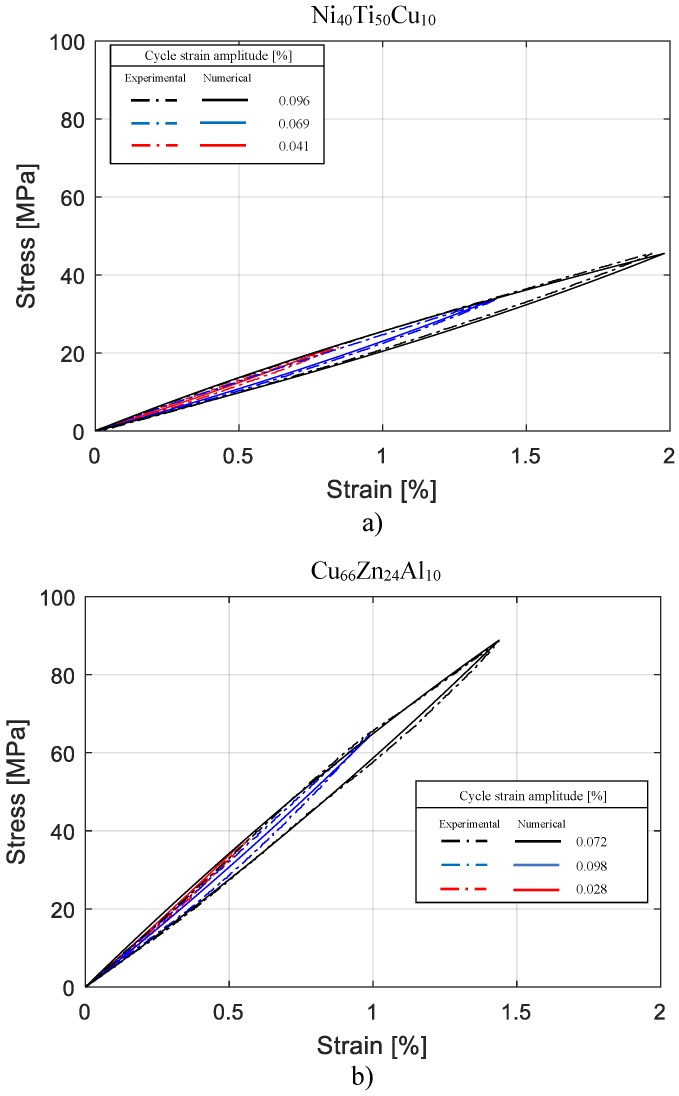
Comparison between experimental and numerical hysteresis cycles: (**a**) Ni_40_Ti_50_Cu_10_; (**b**) Cu_66_Zn_24_Al_10_.

For each composition, the validity of the developed model was proven by comparing the numerical hysteresis loops with additional experimental hysteresis cycles at different amplitudes. The numerical cycles were obtained by simulating, with a plain stress FE model of the specimen, the same condition of experimental cyclic tensile tests explained in the previous section devoted to Materials. The comparison between the experimental and numerical hysteresis loops is reported in Figure 7a for tests on Ni_40_Ti_50_Cu_10_, and in Figure 7b for tests on Cu_66_Zn_24_Al_10_. Three experimental and numerical cycles are represented in each case for comparison. By definition, amplitude of cycle is considered equal to half of maximum stain level of the cycle. A good agreement between the numerical and experimental results is observed. For more comparison, the corresponding loss factor and average elastic modulus of both numerically and experimentally obtained cycles, were calculated and compared. The loss factor corresponding to the hysteresis cycle is given by:

(10) $$\eta =\frac{\Delta U}{2\pi U_{max}}$$

where ΔU is the dissipated energy for each cycle, equal to the enclosed area of the hysteresis cycle, and $U_{max}$ is the maximum elastic energy stored for each cycle. The loss factors calculated for both materials, and for the cycles with different amplitudes, are compared in in Table 3 and Table 4. In both cases, the experimental strain-dependent loss factor of the materials was replicated with a good degree of accuracy.

The corresponding average elastic modulus of each hysteresis cycle was also obtained as:

(11) $$E_{av}=\frac{\sigma_{max}}{\epsilon_{max}}$$

where $\sigma_{max}$ and $\epsilon_{max}$ are the maximum stress and strain of the hysteresis cycles respectively. The average elastic modulus calculated, corresponding to experimental and numerical hysteresis cycles, are compared in Table 3 and Table 4. Obviously, for larger cycles lower values were obtained, but excellent accuracy was achieved. The error was never more than 4%.

## 5. Application to Hybrid Structures

### 5.1. Finite Element Model

In order to show the applicability of the model developed in reproducing an amplitude-dependent damping in dynamic applications, the model was used to model the behaviour of Ni_40_Ti_50_Cu_10_ and Cu_66_Zn_24_Al_10_ SMA layers of a hybrid composite, in the shape of a cantilever beam, in a free decay condition. The free length of the beam was equal to 200 mm, the width amounted to 20 mm and the thickness was 5.2 mm.

**Figure 8:**
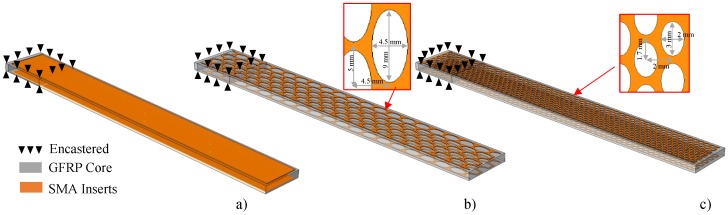
Schematic of a cantilever beam with different hybrid layups. (**a**) Plain; (**b**) Large pattern; (**c**) Small pattern.

The architecture of the hybrid structures is shown in Figure 8. The core composite is a symmetric angle-ply laminated of fiberglass/epoxy resin (3M-SP250 S29A) [+45/−45]_18_ which corresponds to 36 plies with angles of +45 and −45 in alternating sequences. Two layers of SMA alloys can be identified, each 0.2 mm thick, inserted in the matrix glass fiber under the upper and bottom surfaces of the hybrid cantilever beam. Plain and geometrically patterned layers with different dimensions were used, optimized to avoid the delamination of the hybrid composite [41]. Figure 8a shows the hybrid composite with the plain SMA layer, while Figure 8b,c shows the patterned layers with large and small holes respectively. Beside the hybrid layups, the original non-hybrid layup was investigated as a reference in order to compare damping improvements. The original layup is made only from GFRP [+45/−45]_22_ in which SMA sheets are replaced by two plies of GFRP to obtain an almost equivalent thickness. Seven different layups were taken into consideration, as summarized in Table 5. For numerical simulations, seven different layups were reproduced in the Abaqus FE model. The GFRP composite was modelled using 20 node brick elements with a reduced integration. For the hybrid geometry, the thin SMA sheets were modelled with eight node shell elements on the top and bottom surface of the GFRP core. The constraint between the upper and lower surface of the GFRP laminated composite and the thin SMA sheet is a tie constraint. This constraint makes the motion of each node of the SMA sheet equal to the motion of the closest node on the GFRP reference surface. The beam was clamped at one end.

For the hybrid beam model, the SMA-UMAT.for subroutine was used for the SMA layers. For the GFRP core, the Rayleigh damping coefficients were tuned to reproduce the constant damping observed in the experimental test conducted in [12] and an elastic behaviour was assumed. A summary of the material properties is reported in Table 6.

To model the free vibration of the system, in a dynamic implicit step, the free end in the models was subjected to an impulse load. In all of the cases, the Hilber–Hughes–Taylor solver was used which is a common implicit solver in the structural dynamics for the numerical integration and allows for energy dissipation and second order accuracy on contrary to regular Newmark method [42]. The solver parameters were set to: α=−0.005, β=0.275625 and γ=0.55, which guarantees to minimize the added numerical damping while stabilizing the problem. For all of the analyses, a time-increment was set equal to 0.0001 s corresponding to 10 kHz of the sampling rate. This ensured a perfect formation of peaks in a free decay response and guaranteed a more accurate calculation of damping ratios.

The responses of the free ends of the models were recorded, and a transient response was used to measure the damping ratio, using the logarithmic decay method as follows:

(12) $$\zeta =\frac{\delta }{2\pi }=\frac{ln\left(\frac{y_{i}}{y_{i+1}}\right)}{2\pi }$$

where δ is the logarithmic decay and $y_{i}$ corresponds to the magnitude of a peak point in the time decay function, and $y_{i+1}$ corresponds to the magnitude of the peak point one cycle later in the time history.

### 5.2. Results and Discussions

**Figure 9:**
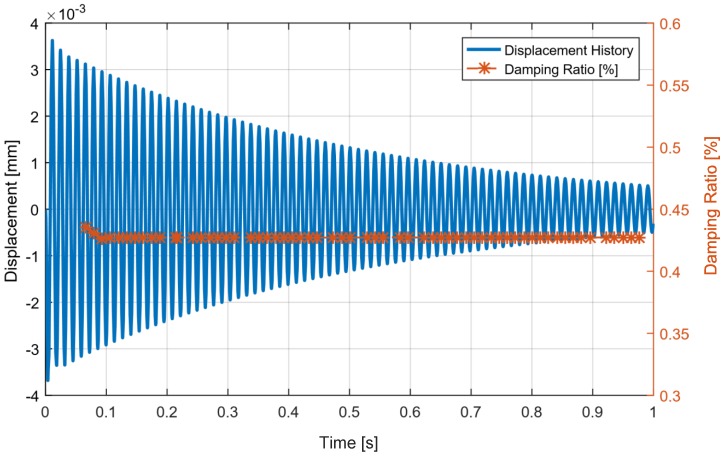
Free decay of a simple beam. A constant damping is reproduced.

**Figure 10:**
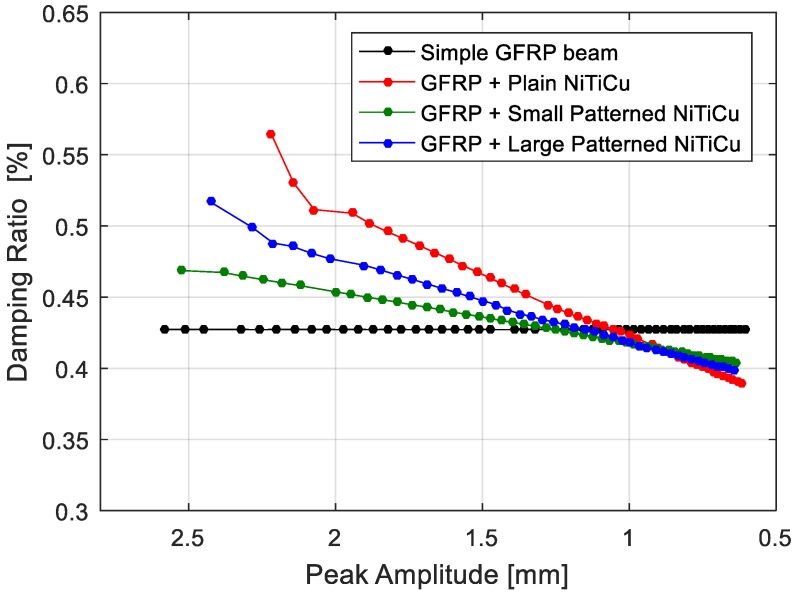
Comparison of the damping ratio as function of displacement for each architecture of Ni_40_Ti_50_Cu_10_ hybrid composite.

**Figure 11:**
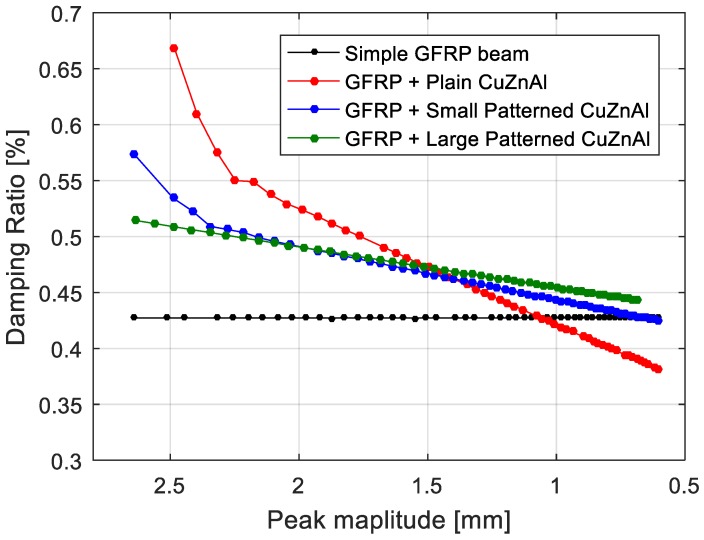
Comparison of the damping ratio as function of displacement for each architecture of Cu_66_Zn_24_Al_10_ hybrid composite.

The free decay of GFRP and hybrid beams are presented in Figure 9. The constant damping was reproduced as expected. The higher damping ratio in the initial peaks relates to presence of higher modes in the vibration of the system. Since the higher modes are damped in a short time, it was not necessary to filter the responses. In the case of hybrid architectures, the amplitude-dependent damping was reproduced successfully by implementing the proposed methodology. Figure 10 and Figure 11 show the comparison of amplitude-dependent damping of the hybrid architectures with the simple beam, respectively, for Ni_40_Ti_50_Cu_10_ and Cu_66_Zn_24_Al_10_ embedded structures.

In the Ni_40_Ti_50_Cu_10_ hybrid beams (Figure 10), it was observed that the damping improvement was more effective at higher amplitudes. This is aligned with the assumption of an amplitude-dependent damping defined for SMA materials. The highest improvement was observed when using the plain sheet. In this case, the rate of the damping ratio change is also higher than the rate observed when a patterned sheet is used, but the drawback of this layup is given by the delamination. For all of the different layups, at a certain point (amplitude less than 1 mm), hybrid structures exhibited lower damping values compared to the simple GFRP beam. This is due to the fact that, Ni_40_Ti_50_Cu_10_ offers a lower damping capacity at low amplitude vibration compared to the GFRP core material.

The Cu_66_Zn_24_Al_10_ hybrid layup follows the same behaviour (Figure 11) reported for the Ni_40_Ti_50_Cu_10_ hybrid layup, while in general damping improvements in general are higher than the one observed previously. The higher improvement lies in the higher average elastic modulus of Cu_66_Zn_24_Al_10_ with respect to Ni_40_Ti_50_Cu_10_. Accordingly, even though the loss factors are slightly less than the reported loss factors reported for Ni_40_Ti_50_Cu_10_, a higher elastic modulus will lead to higher energy dissipation inside the Cu_66_Zn_24_Al_10_ hybrid beams.

## 6. Conclusions

A phenomenological model was developed, by implementing a modified Masing model for an elastic hysteresis behaviour, in order to evaluate the amplitude-dependent damping of Ni_40_Ti_50_Cu_10_ and Cu_66_Zn_24_Al_10_ shape memory alloys in a martensitic state. The hysteresis cycle, for different maximum strain amplitudes, was reproduced numerically for a sample under tensile test and the results were validated with corresponding experimental data.

The validated material model was implemented in a user subroutine of the Abaqus FE Code, and then used to simulate the free decay of the hybrid composite structure in the shape of encastred beams.

The amplitude-dependent damping of the beams was reproduced successfully, confirming that the damping model can be used effectively for accurate numerical simulations of the dynamic behaviour of complex hybrid composite structures.

## Figures and Tables

**Table 1:** Transformation temperatures of the SMA alloys.

Material	$M_{f}$ (°C)	$M_{s}$ (°C)	$A_{s}$ (°C)	$A_{f}$ (°C)
Ni_40_Ti_50_Cu_10_	32	49	52	61
Cu_66_Zn_24_Al_10_	50	63	60	68

**Table 2:** Parameters of the modified Masing model.

Material	$E_{\mathrm{in}}$ (GPa)	$E_{\mathrm{av}}$ (GPa)	$\epsilon_{\max}$ (%)
Ni_40_Ti_50_Cu_10_	28.6	22.26	0.241
Cu_66_Zn_24_Al_10_	66.7	56.9	0.1486

**Table 3:** Comparison of the loss factor and average elastic modulus of Ni_40_Ti_50_Cu_10_, for numerical and experimental hysteresis cycles.

Cycle Strain Amplitude (%)	Loss Factor			Elastic Modulus		
	Numerical (-)	Experimental (-)	Error (%)	Numerical (GPa)	Experimental (GPa)	Error (%)
0.041	0.051	0.049	4.08	26.06	25.24	3.24
0.054	0.064	0.055	16.36	25.20	24.20	4.13
0.069	0.077	0.068	13.23	24.34	24.21	0.53
0.078	0.084	0.082	2.43	23.88	23.87	0.04
0.089	0.091	0.082	10.97	23.38	24.05	2.78
0.096	0.096	0.091	5.49	22.98	23.41	1.83
0.12	0.104	0.106	1.88	22.26	22.29	0.13

**Table 4:** Comparison of the loss factor and average elastic modulus of Cu_66_Zn_24_Al_10_, for numerical and experimental hysteresis cycles.

Cycle Strain Amplitude (%)	Loss Factor			Elastic Modulus		
	Numerical (-)	Experimental (-)	Error (%)	Numerical (GPa)	Experimental (GPa)	Error (%)
0.028	0.025	0.015	66.66	68.06	65.81	3.41
0.049	0.046	0.045	2.22	64.95	65.16	0.32
0.072	0.070	0.072	2.77	61.76	61.57	0.31
0.074	0.073	0.062	17.74	61.43	63.28	2.92

**Table 5:** Layups of the cantilever beams investigated.

Layup No.	SMA Insert	Pattern
Ref Layup	–	
Hyb Layup 1	Ni_40_Ti_50_Cu_10_	–
Hyb Layup 2		Large
Hyb Layup 3		Small
Hyb Layup 4	Cu_66_Zn_24_Al_10_	–
Hyb Layup 5		Large
Hyb Layup 6		Small

**Table 6:** Material properties used in the FEM model.

Material	Elastic Modulus (GPa)	Poisson’s Ratio	Density (kg/m^3^)
GFRP	17	0.27	1880
Ni_40_Ti_50_Cu_10_	Hysteresis model		7400
Cu_66_Zn_24_Al_10_	Hysteresis model		6600
